# Descriptive epidemiology of energy expenditure in the UK: findings from the National Diet and Nutrition Survey 2008–15

**DOI:** 10.1093/ije/dyaa005

**Published:** 2020-03-19

**Authors:** Soren Brage, Tim Lindsay, Michelle Venables, Katrien Wijndaele, Kate Westgate, David Collins, Caireen Roberts, Les Bluck, Nick Wareham, Polly Page

**Affiliations:** MRC Epidemiology Unit, University of Cambridge, Cambridge, UK

**Keywords:** Energy expenditure, physical activity, epidemiology, population

## Abstract

**Background:**

Little is known about population levels of energy expenditure, as national surveillance systems typically employ only crude measures. The National Diet and Nutrition Survey (NDNS) in the UK measured energy expenditure in a 10% subsample by gold-standard doubly labelled water (DLW).

**Methods:**

DLW-subsample participants from the NDNS (383 males, 387 females) aged 4–91 years were recruited between 2008 and 2015 (rolling programme). Height and weight were measured and body-fat percentage estimated by deuterium dilution.

**Results:**

Absolute total energy expenditure (TEE) increased steadily throughout childhood, ranging from 6.2 and 7.2 MJ/day in 4- to 7-year-olds to 9.7 and 11.7 MJ/day for 14- to 16-year-old girls and boys, respectively. TEE peaked in 17- to 27-year-old women (10.7 MJ/day) and 28- to 43-year-old men (14.4 MJ/day), before decreasing gradually in old age. Physical-activity energy expenditure (PAEE) declined steadily with age from childhood (87 kJ/day/kg in 4- to 7-year-olds) through to old age (38 kJ/day/kg in 71- to 91-year-olds). No differences were observed by time, region and macronutrient composition. Body-fat percentage was strongly inversely associated with PAEE throughout life, irrespective of expressing PAEE relative to body mass or fat-free mass. Compared with females with <30% body fat, females with >40% recorded 29 kJ/day/kg body mass and 18 kJ/day/kg fat-free mass less PAEE in analyses adjusted for age, geographical region and time of assessment. Similarly, compared with males with <25% body fat, males with >35% recorded 26 kJ/day/kg body mass and 10 kJ/day/kg fat-free mass less PAEE.

**Conclusions:**

This first nationally representative study reports levels of human-energy expenditure as measured by gold-standard methodology; values may serve as a reference for other population studies. Age, sex and body composition are the main determinants of energy expenditure.

Key Messages

- This is the first nationally representative study of human energy expenditure, covering the UK in the period 2008-2015.
- Total energy expenditure (MJ/day) increases steadily with age throughout childhood and adolescence, peaks in the 3^rd^ decade of life in women and 4^th^ decade of life in men, before decreasing gradually in old age.
- Physical activity energy expenditure (kJ/day/kg or kJ/day/kg fat-free mass) declines steadily with age from childhood to old age, more steeply so in males.
- Body-fat percentage is strongly inversely associated with physical activity energy expenditure.
- We found little evidence that energy expenditure varied by geographical region, over time, or by dietary macronutrient composition.

## Introduction

Little is known about population levels of energy expenditure (EE), as most national surveys use proxy methods for assessment—typically questionnaires. These may take the form of either self-reported dietary energy intake combined with measures of weight change^1^ or self-reported physical activity combined with estimates of resting EE.^2^ The former approach is challenged not only by the necessary correction for any weight changes, but also by possible under-reporting of energy intake by overweight or obese individuals.^3^ The latter approach does not need to make assumptions about energy balance, as it is directly assessing the expenditure side; however, self-report methods for physical activity also have limited accuracy, and this applies particularly to derivatives such as estimates of EE.^4^ The use of objective methods in the form of wearable sensors such as accelerometers and heart-rate monitors is typically preferred as the objective methods for large-scale population studies, since these provide information about intensity patterns as well as more precise estimates of EE when coupled with appropriate inference models.^5–9^ Irrespective of the success of such inference models, feasibility is somewhat limited for methods using heart-rate monitoring due to its requirement for individual calibration using an exercise test,^10^^,^^11^ whereas the main limitation of accelerometry-based estimation of EE depends on the mix of specific behaviours in which the population under study is engaged, as this relationship varies by activity type.^12^^,^^13^

Preferably, one would therefore employ more direct, yet highly feasible, measurements of the quantity of interest for the surveillance of population trends in EE. The doubly labelled water (DLW) technique is the gold standard for measurement of EE during free-living.^14^ This technique uses the stable isotopes deuterium (^2^H) and Oxygen-18 (^18^O) to directly measure the rate of carbon-dioxide production (rCO_2_) over a period of 1–2 weeks, from which the average total energy expenditure (TEE) can be calculated with high precision. Combined with simple anthropometric measurements, estimates of physical-activity energy expenditure (PAEE) can also be derived. The DLW method is highly feasible in terms of low participant burden but it is unfortunately also expensive and hence is only seldom used in large studies.

The National Diet and Nutrition Survey (NDNS) employs a nationally representative sampling frame to assess the diet and nutritional status of the general population aged 1.5 years or older living in private households in the UK.^15^ One of the unique features of the NDNS is that a 10% subsample of all age groups 4 years or older also had EE assessed using the DLW technique over 10 days of free-living. The aim of this study was to describe the variation in components of EE by key personal characteristics, geographical location and over time.

## Methods

### Participants

This is a repeat cross-sectional survey. Participants were recruited to the rolling programme in the NDNS by stratified and clustered random sampling of households in the UK. NDNS data are weighted to account for any selection or response biases to ensure results are representative of the UK population.^15^ A total of 15 583 households were selected to take part between 2008 and 2015, and 8974 households agreed (58% household response rate). From those households, 10 727 individuals agreed to take part and a subsample of these NDNS participants were invited to take part in the DLW sub-study, within which individuals were sampled according to pre-specified age/sex strata (4–10, 11–15, 16–49, 50–64, and 65+ years). The DLW sub-study fieldwork was carried out in two waves; for the first wave (2008–11), targets were 40 participants in each of the age/sex groups but, for the second wave (2013–15), these were changed to 30 participants for each stratum for those aged 4–10 and those 65+ years, and to 50 participants for those aged 16–49 years. A total of 808 were invited to take part in the DLW sub-study, of whom 770 participants provided sufficient data to derive valid EE estimates and they constitute the sample included in the present analysis. This subsample does not differ from the main NDNS (excluding children <4 years) in terms of sex, body mass index (BMI), total energy intake, fruit-and-vegetable intake in g/day, free-sugar intake (% total energy intake) and saturated-fat intake (% total energy intake) but it was 2.6 years older.^15^

All adult participants provided informed written consent and all children provided assent with written consent from their legal guardian. The study was approved by the Oxfordshire A Research Ethics Committee (#07/H0604/113) and Cambridge South NRES Committee (#13/EE/0016).

### Measurements

Anthropometric measurements were performed in the participants’ homes. Height was measured to the nearest millimetre using a portable stadiometer and body mass was measured to the nearest 100 g in light clothing using calibrated scales.^15^ The BMI (kg/m^2^) was calculated from these measures.

Food-and-drink intake was captured using a 4-day unweighted (estimated) paper diary. Average nutrient intakes were calculated using DINO (Diet In, Nutrients Out),^16^ which incorporates Public Health England’s NDNS nutrient databank, from which total energy intake and macronutrient composition (fat, carbohydrate, protein and alcohol) were determined. This method was selected following a comparison study prior to the start of the NDNS Rolling Programme that evaluated two candidate methods: the estimated food diary and the repeat 24-hour recall. Both methods were feasible and provided similar information on food, energy and nutrient intake. The diary was selected based on continuity with past NDNS surveys and flexibility with a wide range of age groups.^17^

For the measurement of TEE, a baseline (pre-dose) urine sample was first collected to establish the natural abundance of the ^2^H and ^18^O isotopes in body water. Next, a dose of ^2^$H_{2}^{18}$O proportional to the participant’s body mass (80 mg per kg body mass of ^2^H_2_O and 150 mg per kg body mass of $H_{2}^{18}$O) was prepared in a dose bottle. The full dose was drunk using a straw, following which the bottle was refilled with local tap water and again fully drunk by the participant. Participants collected single daily spot urine samples for the next 10 days, representing about 2.5 half-lives of peak enrichment. The urine samples were analysed for isotopic enrichment by mass spectrometry (^18^O enrichment: AP2003, Analytical Precision Ltd, Northwich, Cheshire, UK; ^2^H enrichment: Isoprime, GV Instruments, Wythenshaw, Manchester, UK or Sercon ABCA-Hydra 20–22, Sercon Ltd, Crewe, UK). The rate of carbon-dioxide production was measured using the method of Schoeller^18^ and converted to TEE using the energy equivalents of CO_2_ of Elia and Livesey^19^ using the food quotient as an approximation of the respiratory exchange quotient. Total body water was assessed using the zero-time intercept of deuterium turnover^20^ and fat-free body mass calculated using a hydration factor of 73%.^21^ Body-fat percentage was calculated as total body mass minus fat-free mass, expressed as a percentage of the total.

The resting metabolic rate was estimated from anthropometry variables by averaging three prediction equations: one based on age, sex, height and total body mass derived in a large database^22^ and two based on smaller studies that also take into account body composition.^23^^,^^24^ In order to calculate the 24-hour resting energy expenditure (REE), we integrated this resting metabolic rate value over time, but with a small adjustment for the 5% lower metabolic rate observed during sleep,^25^ applied using age-specific sleep durations ranging from 8 to 12 hours/day.^26^ The diet-induced thermogenesis (DIT) was calculated from the macronutrient composition of the diet as previously described^7^^,^^27^ and the PAEE was calculated as the residual EE, which sums REE and DIT to make up TEE, according to the equation PAEE = TEE − REE − DIT.

### Statistics

We expressed the daily TEE in absolute units (MJ/day) and both TEE and PAEE in relative units (kJ/day/kg body mass). In sensitivity analyses, we also expressed EE in units scaled to fat-free body mass and in allometrically scaled units of kJ/day/kg^2/3^ body mass, the latter based on the theoretical principle that absolute EE scales to bodily dimensions to the power of 2 and body-mass scales to bodily dimensions to the power of 3.^28^^,^^29^ We present the summary statistics (mean and standard deviation) of all estimates of EE by recruitment strata, i.e. age and sex groups. In addition, we present box plots (box denoting median and interquartile ranges) by expanded age groups (deciles), as well as by survey year (2008–11 and 2012–15) and main geographical regions of North England, South England and Scotland/Wales/North Ireland combined. North England included the following Government Office Regions; North East, North West, Yorkshire and The Humber, East Midlands and West Midlands; and South England comprised the East, South West, London and South East, as used previously.^30^ We examine the association with obesity status by both BMI and body-fat groups, stratified by sex and age groups. To examine independent associations, we performed a multiple linear-regression analysis with mutual adjustment for all above factors and with additional adjustment for season of measurement [expressed as two orthogonal sine functions: ‘winter’ (with max = 1 on 1^st^ January and min = −1 on 1^st^ July) and ‘spring’ (with max = 1 on 1^st^ April and min = −1 on 1^st^ October)]. A sensitivity analysis of the BMI association was performed using fat mass index (FMI) and fat-free mass index (FFMI) in age- and sex-specific tertiles, and finally a supplementary analysis describing EE by macronutrient-composition groups was performed to investigate possible behavioural associations.

## Results

Of the 770 participants with valid DLW data included in this analysis, the four constituent countries of the UK were represented with 568 participants from England, 50 from Scotland, 72 from Wales and 80 from Northern Ireland (Table 1).

**Table 1.** Participant characteristics

	Females					Males				
Age group	4–10	11–15	16–49	50–64	65–91	4–10	11–15	16–49	50–64	65–91
Age (years)	7.5 (2)	13.4 (1)	31.9 (11)	57.0 (5)	72.9 (6)	7.1 (2)	12.8 (1)	29.2 (11)	56.4 (5)	73.3 (6)
*N*	73	80	91	79	64	74	76	89	83	61
Survey year (*n*)										
2008–11	41	38	40	37	32	41	34	38	41	29
2013–15	32	42	51	42	32	33	42	51	42	32
Region (*n*)										
South England	28	24	28	26	25	23	26	23	24	20
North England	31	34	36	32	23	34	23	41	44	23
Scotland	4	9	4	6	5	4	5	3	5	5
Wales	4	6	7	8	9	6	11	9	5	7
North Ireland	6	7	16	7	2	7	11	13	5	6
Anthropometry										
Height (cm)	127 (13)	159 (8)	164 (7)	162 (6)	160 (7)	126 (11)	159 (10)	178 (6)	175 (7)	172 (6)
Weight (kg)	28.5 (9)	54.7 (13)	70.0 (17)	76.5 (16)	73.5 (14)	26.6 (6)	53.0 (13)	82.7 (19)	86.7 (15)	82.8 (14)
BMI (kg/m^2^)	17.2 (3)	21.4 (4)	26.2 (6)	29.3 (6)	28.7 (5)	16.6 (2)	20.6 (4)	26.2 (6)	28.2 (4)	28.0 (4)
FFMI (kg/m^2^)	12.6 (1)	14.4 (2)	16.1 (2)	16.4 (2)	16.0 (2)	13.0 (1)	15.1 (2)	18.7 (3)	19.1 (2)	18.2 (2)
FMI (kg/m^2^)	4.6 (2)	7.0 (3)	10.1 (5)	12.9 (4)	12.7 (4)	3.6 (2)	5.6 (3)	7.5 (4)	9.1 (3)	9.8 (3)
Body fat (%)	26 (7)	31 (8)	37 (8)	43 (6)	43 (6)	21 (7)	26 (9)	27 (9)	32 (7)	34 (7)
Diet										
Carbohydrate intake (% energy)	54 (4)	52 (5)	49 (8)	46 (7)	47 (7)	53 (4)	54 (5)	49 (7)	47 (7)	46 (7)
Fat intake (% energy)	32 (4)	34 (4)	32 (6)	33 (6)	34 (6)	33 (4)	32 (4)	32 (6)	32 (6)	33 (6)
Protein intake (% energy)	14 (2)	14 (3)	15 (4)	16 (4)	17 (3)	14 (2)	14 (3)	15 (4)	16 (3)	16 (3)
Alcohol intake (% energy)	0 (.0)	0 (.4)	3 (7)	4 (5)	2 (4)	0 (.0)	0 (.0)	4 (7)	6 (7)	5 (6)
Energy expenditure										
Diet-induced thermogenesis (MJ/d)	.6 (.1)	.8 (.1)	1.0 (.2)	1.0 (.2)	.9 (.2)	.7 (.1)	1.0 (.2)	1.3 (.3)	1.2 (.3)	1.1 (.3)
REE (MJ/d)	4.3 (.5)	5.6 (.6)	6.0 (.7)	5.8 (.6)	5.3 (.6)	4.5 (.4)	6.1 (.8)	7.5 (.9)	7.1 (.8)	6.5 (.7)
TEE (MJ/d)	7.2 (1)	9.8 (2)	10.8 (2)	10.3 (1)	9.2 (1)	7.7 (1)	11.3 (2)	13.9 (3)	13.0 (2)	11.4 (2)
TEE (kJ/d/kg)	263 (42)	185 (33)	158 (29)	138 (25)	127 (23)	300 (47)	221 (40)	173 (32)	152 (25)	138 (22)
PAEE (kJ/d/kg)	82 (19)	64 (21)	56 (21)	48 (18)	42 (16)	99 (31)	84 (27)	64 (24)	55 (20)	46 (17)

National Diet and Nutrition Survey doubly labelled water subsample (2008–15).

Data are *N* or mean (SD). BMI, body mass index; FFMI, fat-free mass index; FMI, fat mass index; REE, resting energy expenditure; TEE, total energy expenditure; PAEE, physical-activity energy expenditure.

The mean (SD) TEE was 10.6 (2.8) MJ/day or 185 (63) kJ/day/kg, REE was 5.9 (1.2) MJ/day and PAEE was 64 (28) kJ/day/kg. Across these estimates of EE, after adjustment for age, time and region of measurement, male sex was associated with higher values (*p* < 0.001). When TEE and PAEE were expressed relative to fat-free mass, only PAEE was higher in males (*p* = 0.010).

**Figure 1.**
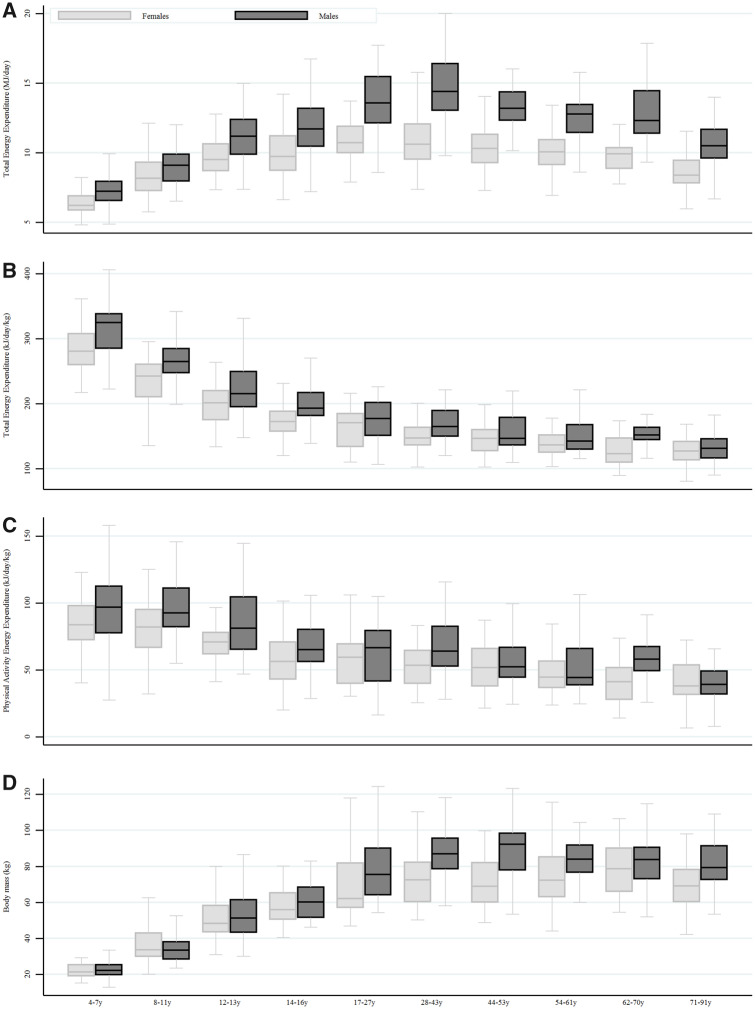
Total (panels **A** and **B**) and physical activity-related (panel **C**) energy expenditure by age (approximate deciles) and sex groups (females = light grey; males = dark grey). Bottom panel (**D**) shows stratified body mass.

Figure 1 shows TEE, PAEE and body mass across age deciles and stratified by sex. Median TEE and PAEE were higher in males than in females across all age groups; body mass was similar in boys and girls up to age 16 years but higher in men above that age. Absolute TEE (MJ/day) was highest in 17- to 27-year-old women and 28- to 43-year-old men, respectively. In contrast, TEE and PAEE relative to body mass (kJ/day/kg) was highest in the youngest individuals and displayed a consistent downward trend with advancing age into adulthood. TEE had a less steep association with age from early to later adulthood. The body-mass-scaled EE associations partially mirrored the positive trend in body mass from childhood into young adulthood, which levelled off across adult ages. Similar age associations were observed in the sensitivity analyses (Supplementary Figures 1 and 2, available as Supplementary data at *IJE* online), although the age association for allometrically scaled TEE (kJ/day/kg^2/3^) was more linear across the whole age range and 8- to 11-year-olds had the highest PAEE of all groups in these analyses.

**Figure 2.**
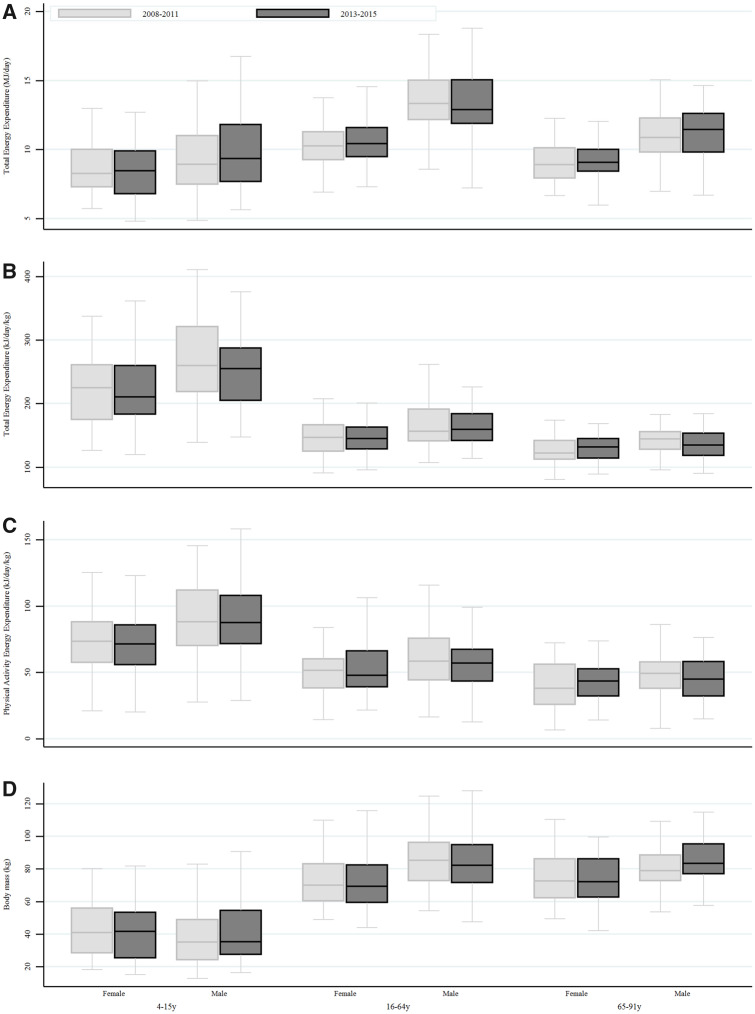
Age- and sex-specific total (panels **A** and **B**) and physical-activity-related (panel **C**) energy expenditure by survey year (2008–11 = light grey; 2013–15 = dark grey). Bottom panel (**D**) shows stratified body mass.

**Figure 3.**
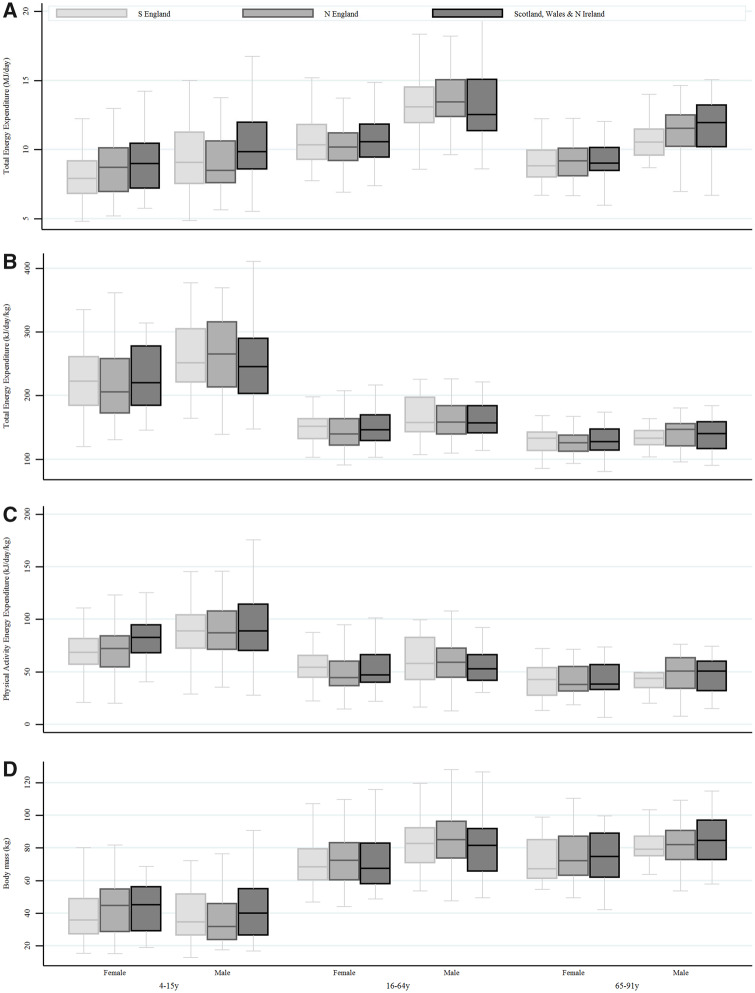
Age- and sex-specific total (panels **A** and **B**) and physical-activity-related (panel **C**) energy expenditure by geographical region (South England = light grey; North England = medium grey; Scotland, Wales and North Ireland = dark grey). Bottom panel (**D**) shows stratified body mass.

There were no significant differences in TEE, PAEE or body mass among those participants surveyed between 2008 and 2011 and those surveyed between 2013 and 2015 (Figure 2), nor were there any discernible differences between constituent geographical regions (Figure 3). These observations were confirmed in the multivariable adjusted analyses, which were additionally adjusted for season of measurement—an effect that was only apparent in males, with slightly higher values in spring (Table 2).

**Table 2.** Mutually adjusted associations with energy expenditure. National Diet and Nutrition Survey doubly labelled water subsample (2008–15)

	Total energy expenditure (MJ/day)	95% CI	Total energy expenditure (kJ/day/kg)	95% CI	Physical-activity energy expenditure (kJ/day/ kg)	95% CI
Females						
Age (years)						
4–10	Reference		Reference		Reference	
11–15	2.41***	1.92; 2.90	–73.3***	–82.0; –64.6	–17.1***	–22.9; –11.2
16–49	3.07***	2.58; 3.57	–91.2***	–100.0; –82.4	–20.6***	–26.5; –14.7
50–64	2.08***	1.52; 2.64	–98.5***	–108.4; –88.5	–23.1***	–29.7; –16.4
65–91	1.05***	0.47; 1.62	–110.8***	–121.0; –100.6	–30.5***	–37.3; –23.7
Year of study						
2008–11	Reference		Reference		Reference	
2013–15	0.03	–0.28; 0.34	3.5	–2.0; 9.0	1.0	–2.7; 4.7
Season						
Spring	0.02	–0.20; 0.24	–0.1	–4.0; 3.7	0.0	–2.6; 2.6
Winter	0.10	–0.12; 0.33	0.2	–3.7; 4.2	0.9	–1.7; 3.6
Region						
South England	Reference		Reference		Reference	
North England	–0.08	–0.43; 0.28	–3.4	–9.7; 2.9	–1.6	–5.8; 2.6
Scotland, Wales, Northern Ireland	0.16	–0.24; 0.56	5.0	–2.1; 12.1	3.9	–0.9; 8.6
BMI category						
<25 kg/m^2^	Reference		Reference		Reference	
25–30 kg/m^2^	0.73***	0.30; 1.15	–30.2***	–37.8; –22.6	–12.8***	–17.9; –7.7
>30 kg/m^2^	1.78***	1.32; 2.23	–43.2***	–51.3; –35.2	–18.8***	–24.2; –13.4
Constant	7.16***	6.74; 7.58	262.6***	255.1; 270.0	82.4***	77.4; 87.4
Model 2 (BF% instead of BMI category)						
F: <30% M: <25%	Reference		Reference		Reference	
F: 30–40% M: 25–35%	0.09	–0.39; 0.57	–31.1***	–38.3; –23.9	–14.3***	–19.3; –9.3
F: >40% M: >35%	0.71***	0.20; 1.23	–61.2***	–68.9; –53.6	–29.0***	–34.3; –23.7
Model 3 (FFMI instead of BMI category)						
Tertile 1	Reference		Reference		Reference	
Tertile 2	0.92***	0.60; 1.25	–13.9***	–21.3; –6.5	–1.4	–6.2; 3.4
Tertile 3	2.50***	2.16; 2.84	–24.6***	–32.4; –16.8	–2.1	–7.1; 3.0

	Total energy expenditure (MJ/day)	95% CI	Total energy expenditure (kJ/day/kg)	95% CI	Physical-activity energy expenditure (kJ/day/kg)	95% CI

Males						
Age (years)						
4–10	Reference		Reference		Reference	
11–15	3.27***	2.62; 3.91	–75.0***	–85.6; –64.4	–13.7***	–21.5; –5.8
16–49	5.03***	4.36; 5.71	–107.7***	–118.8; –96.5	–27.1***	–35.4; –18.8
50–64	3.56***	2.81; 4.31	–122.1***	–134.4; –109.7	–33.8***	–42.9; –24.7
65–91	1.80***	0.99; 2.61	–135.4***	–148.8; –122.1	–42.7***	–52.5; –32.8
Year of study						
2008–11	Reference		Reference		Reference	
2013–15	–0.16	–0.57; 0.25	–4.1	–10.9; 2.7	–3.0	–8.0; 2.1
Season						
Spring	0.36**	0.09; 0.64	2.1	–2.5; 6.7	2.7	–0.7; 6.1
Winter	0.20	–0.10; 0.50	–0.2	–5.1; 4.8	–0.5	–4.1; 3.2
Region						
South England	Reference		Reference		Reference	
North England	0.21	–0.26; 0.69	–1.1	–8.9; 6.7	0.0	–5.7; 5.8
Scotland, Wales, Northern Ireland	0.21	–0.33; 0.75	3.5	–5.3; 12.3	4.3	–2.3; 10.8
BMI Category						
<25 kg/m^2^	Reference		Reference		Reference	
25–30 kg/m^2^	1.49***	0.93; 2.06	–27.6***	–36.9; –18.3	–11.0***	–17.9; –4.1
>30 kg/m^2^	2.87***	2.22; 3.52	–40.8***	–51.5; –30.1	–15.8***	–23.7; –7.9
Constant	7.74***	7.18; 8.31	301.7***	292.5; 311.0	99.4***	92.5; 106.2
Model 2 (BF% instead of BMI category)						
F: <30% M: <25%	Reference		Reference		Reference	
F: 30–40% M: 25–35%	0.57**	0.03; 1.10	–36.1***	–43.4; –28.8	–16.3***	–22.1; –10.6
F: >40% M: >35%	0.90***	0.27; 1.53	–54.5***	–63.0; –46.0	–25.6***	–32.3; –18.9
Model 3 (FFMI instead of BMI category)						
Tertile 1	Reference		Reference		Reference	
Tertile 2	1.47***	1.02; 1.92	–12.95***	–21.39; –4.52	1.28	–4.77; 7.32
Tertile 3	2.96***	2.48; 3.44	–17.35***	–26.31; –8.39	1.89	–4.53; 8.31

BMI, body mass index; BF%, body-fat percentage; FFMI, fat-free mass index; PAEE, physical-activity energy expenditure; CI, confidence interval.

*** *p* < 0.01;

** *p* < 0.05;

* *p* < 0.1.

**Figure 4:**
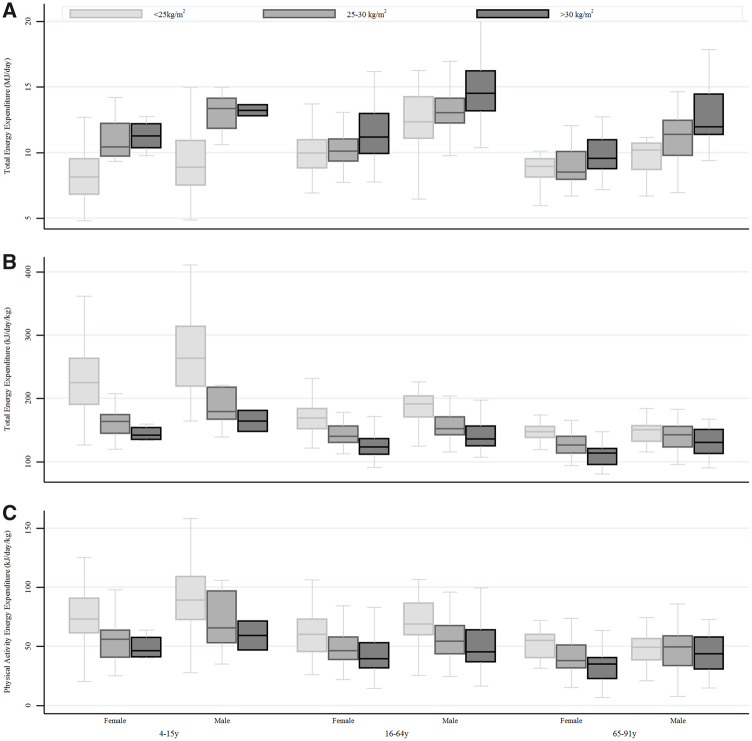
Age- and sex-specific total (panels **A** and **B**) and physical-activity-related (panel **C**) energy expenditure by body mass index category [normal-weight (<25 kg/m^2^) = light grey; overweight (25–30 kg/m^2^) = medium grey; obese (>30 kg/m^2^) = dark grey].

Across the sample, absolute TEE (MJ/day) was higher in individuals with higher BMI. Overweight participants had higher TEE (MJ/day) than normal-weight participants and obese participants accumulated higher TEE levels than overweight participants—a trend that was observed within nearly all age and sex strata (Figure 4). However, this relationship was inverse when TEE was expressed in relative terms. Obese males and females in all age groups recorded the lowest relative TEE and PAEE (kJ/day/kg), whereas normal-weight individuals recorded the highest.

**Figure 5:**
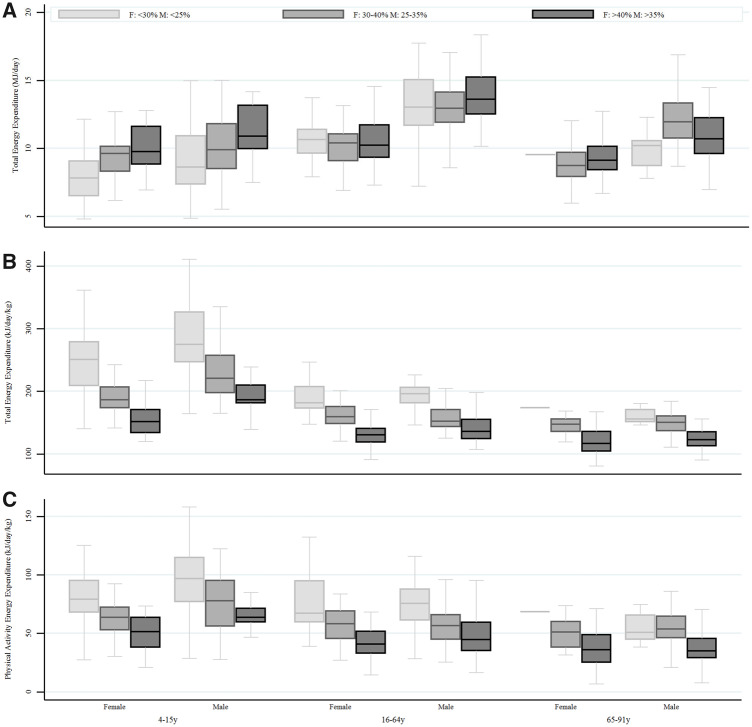
Age- and sex-specific total (panels **A** and **B**) and physical-activity-related (panel **C**) energy expenditure by body-fat percentage groups (slimmest = light grey; medium body composition = medium grey; fattest = dark grey).

A similar relationship was also observed for TEE and PAEE across groups of differing body-fat percentage, although the clear positive trend for absolute TEE was absent in the two adult age groups (Figure 5). For relative TEE and PAEE (kJ/day/kg), those with the highest body-fat percentage recorded the lowest EE, whereas the slimmest individuals recorded the highest. The sole exception to this were men aged 65–91 years with medium body fat who, as a group, accumulated slightly more PAEE than their slimmer counterparts. The multivariable regression analysis confirmed associations with BMI and body fatness in both sexes (Table 2). FFMI, however, was only positively associated with absolute TEE and inversely associated with relative TEE, but not related to relative PAEE in either males or females.

In sensitivity analyses modelling PAEE per kg fat-free mass (Supplement Table 1, available as Supplementary data at *IJE* online), individuals in the third tertile of FMI were less active; this inverse association was also observed for body-fat-percentage groups. This sensitivity analysis also suggested a possible regional difference in activity levels, with women in Wales, Scotland and Northern Ireland expending more activity energy per kg fat-free mass, independently of other covariates.

**Figure 6:**
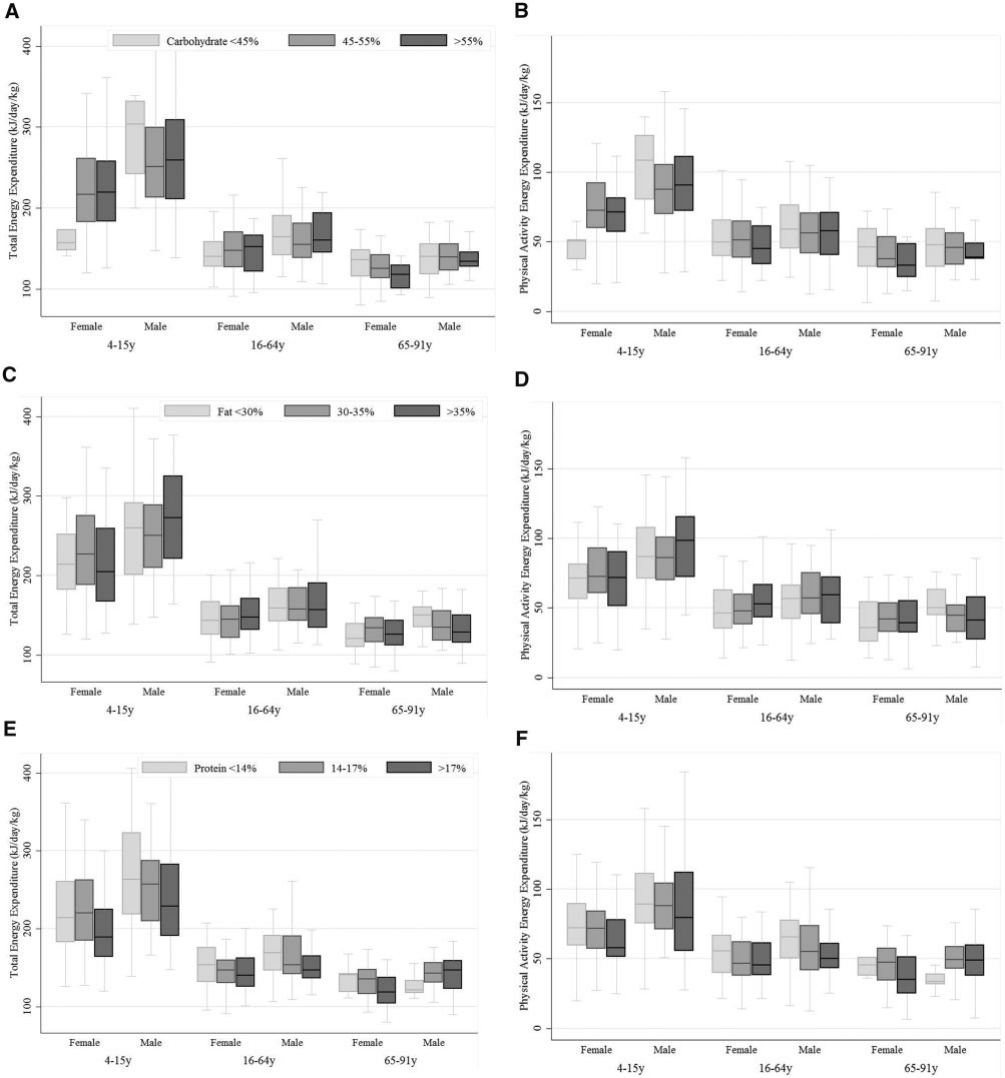
Total (left panels) and physical-activity-related (right panels) energy expenditure by dietary-intake groups of carbohydrate (<45%, 45–55%, >55% energy, top panels **A** and **B**), fat (<30%, 30–35%, >35% energy, middle panels **C** and **D**) and protein (<14%, 14–17%, >17% energy, bottom panels **E** and **F**), stratified by age and sex groups.

Associations between macronutrient composition and EE were generally weak but trending towards higher PAEE in groups consuming a lower proportion of their energy intake from carbohydrate or protein. However, young girls who consumed low-carbohydrate diets were less active than their counterparts, as were older men who consumed low-protein diets (Figure 6).

## Discussion

Here, we report gold-standard-measured EE from a nationally representative cross-sectional UK survey. Our results show how TEE and PAEE vary according to age, sex and body composition but no differences were observed by geographical region of the UK or over time in the period between 2008 and 2015.

Our results demonstrate that males accumulate higher overall levels of TEE and PAEE than females across all ages—a finding that is consistent with other British cohort studies investigating energy expenditure by objective methods.^31–36^ Age was an important correlate of PAEE and TEE in both sexes, with similar patterns across the lifespan for all EE measures; absolute TEE peaks in the early adult years, before dropping off around retirement age, whereas relative TEE and PAEE are highest in the earliest years of life before gradually declining steeply at first and reflecting in part natural growth and development, and then more shallowly after the age at which adult height is typically attained.

There are no previous reports of nationally representative DLW-based estimates of EE from the UK, or from other countries. However, within the UK, some preparatory NDNS work in 1989 measuring 81 children from Cambridgeshire aged 1.5–4.5 years old reported TEE levels of 4.9 MJ/day (333 kJ/day/kg) in the whole group and 5.4 MJ/day (320 kJ/day/kg) in 4-year-olds (*n* = 27).^35^ Assuming a DIT of 10% TEE and estimating REE^22^^,^^26^ suggests PAEE around 77 kJ/day/kg (79 kJ/day/kg for 4-year-olds). The 2008–15 NDNS DLW subsample does not include children younger than 4 years but the eight 4-year-old children included had TEE of 5.9 MJ/day (314 kJ/day/kg) and PAEE of 86 kJ/day/kg.

A sample of 78 children aged 3–18 years from Belfast (Northern Ireland) measured in 1991 (or earlier) had TEE values of 7.1 MJ/day (313 kJ/day/kg) in 3- to 10-year-olds (*n* = 44) and 11.8 MJ/day (211 kJ/day/kg) in 12- to 18-year-olds (*n* = 34)^36^; estimating PAEE as above yields values of 81 and 108 kJ/day/kg in 3- to 10-year-old girls and boys, and 67 and 90 kJ/day/kg in 12- to 18-year-old girls and boys, respectively.

Overall, these historical UK estimates of EE are not very different to those we report here from the most recent NDNS survey but no firm conclusions on secular trends in EE in UK children can be drawn owing to regional differences in population sampling and small sample sizes.

Considering more contemporary UK data, 1397 British 6-year-olds measured by combined heart rate and movement sensing recorded PAEE of 95 kJ/day/kg, which is almost identical to NDNS values.^37^ In fact, several British cohort studies using this technique observe comparable PAEE levels across the age range, with 66 and 84 kJ/day/kg in 825 adolescent girls and boys (aged 15 years) attending schools in Cambridge^31^ and 50 and 59 kJ/day/kg in a sample of 12 002 English adult women and men aged between 29 and 64 years (mean 49 years)^34^; the latter cohort also reported a DLW-measured PAEE of 50 kJ/day/kg in a subsample of 100 men and women (mean age 54 years).^9^ In older adults, PAEE by combined sensing was reported as 34 and 36 kJ/day/kg in 1787 women and men of the nationally representative UK 1946 birth cohort assessed at ages 60–64 years^33^; this compares to PAEE of 32 kJ/day/kg observed in 23 Cambridge men aged 76–88 years measured some time before 1995, with TEE of 9.2 MJ/day (129 kJ/day/kg).^38^

Internationally, only limited DLW data are available from large single studies but pooled analyses from multiple smaller studies have been reported. Torun reported TEE of 7.5 MJ/day (259 kJ/day/kg) in 657 girls and 8.0 MJ/day (287 kJ/day/kg) in 483 boys aged 1–18 years, including data from UK studies reported above; the remaining studies were mostly from North America, followed by Northern Europe and Latin America.^39^ Comparing estimates from Latin America vs other countries, TEE was 6.5 vs 6.7 MJ/day (292 vs 275 kJ/day/kg) in 3- to 11 year-old girls and 7.1 vs 7.2 MJ/day (295 vs 296 kJ/day/kg) in boys, respectively. In parallel, PAEE was 86 vs 77 kJ/day/kg in girls and 87 vs 86 kJ/day/kg in boys. In older children, TEE was 10.5 MJ/day (193 kJ/day/kg) in girls and 13.0 MJ/day (225 kJ/day/kg) in boys, and PAEE was 71 and 84 kJ/day/kg, respectively; these estimates did not include any studies from Latin America. Combined, these studies show similar TEE but varying levels of PAEE, although direct comparisons should bear in mind notable differences between studies, including participant selection, setting and era.

In adults, male NDNS participants accumulated a mean TEE and PAEE of 12.9 MJ/day and 55 kJ/day/kg, whereas adult women accumulated 10.1 MJ/day and 48 kJ/day/kg, respectively. This is comparable to levels of TEE and PAEE in other DLW studies in comparable populations, e.g. mean TEE of 12.7 MJ/day for men and 10.0 MJ/day for women, and PAEE of approximately 54 and 44 kJ/day/kg were reported in a meta-analysis of 1575 men and 2914 women aged over 19 years from high-development index countries.^40^ This analysis included published DLW data up to 2011 and, although studies in special populations were excluded, again caution is warranted as to the representativeness of the participants included.

More recently, Matthews *et al.* reported DLW results from a study of 461 American men and 471 women in a convenience sample with mean ages of 64 and 62 years, respectively. In that study, mean TEE was 11.6 and 9.1 MJ/day and mean PAEE was 39 and 38 kJ/day/kg for men and women, respectively.^41^ Again, these figures are very similar to those found for TEE in the oldest category of NDNS participants (>64 years) and only about 10% lower for PAEE, although we note the mean age was 72 years in our sample. Overall, the results therefore suggest that British men and women expend a similar amount of total and physical-activity energy to their counterparts in the developed world, with a similar age-related decline.

This is partially in contrast to EE levels in populations residing in less developed countries, where only absolute EE levels are similar but activity levels are higher. For example, absolute TEE in studies from countries with low-to-medium development scores was reported to be 12.3 and 9.3 MJ/day, but relative PAEE estimated at 69 and 49 kJ/day/kg, for men and women, respectively.^40^ With respect to PAEE, these high levels seem particularly pronounced in rural dwellers in these countries, with values of ∼60 kJ/day/kg in Cameroon^42^ and even higher in rural Luo, Kamba and Masai in Kenya^43^ as assessed with individually calibrated combined heart-rate and movement sensing.

BMI and body-fat percentage were also important correlates of TEE and PAEE, and there is an ongoing debate over how to best express EE with respect to body size, particularly when examining associations with overweight and obesity.^44^^,^^45^ In the NDNS sample, larger body size was associated with higher absolute levels of TEE (MJ/day) but, irrespective of how EE was expressed relative to body weight (kJ/day/kg or kJ/day/kg^2/3^), BMI displayed an inverse relationship. This was also observed when body fatness was assessed in terms of total body-fat percentage or FMI. Multivariate analysis demonstrated that, when corrected for age, geographical region, survey year and season of measurement, overweight women accumulated 30 kJ/day/kg less TEE and 13 kJ/day/kg less PAEE than their normal-BMI counterparts. Continuing this trend, obese females accumulated 43 kJ/day/kg less TEE and 19 kJ/day/kg less PAEE than normal-weight female participants. This was replicated in males with TEE and PAEE lower in groups with higher BMI.

This finding highlights the role that absolute body size plays in the accumulation of absolute EE, but also underlines obesity’s inverse association with PAEE. This relationship was apparent regardless of the measure of obesity and of the measure of physical activity, with those with higher absolute body-fat levels and those in the highest FMI category accumulating less physical activity than slimmer counterparts. However, the association with FFMI was non-significant in both males and females, as also observed in a pooled analysis of 529 Dutch adults,^46^ many of whom were included in the meta-analysis by Dugas *et al.*, who reported non-significant associations between physical-activity level (TEE/REE) and body weight.^40^ This highlights the complex interplay between physical activity, EE, body mass and diet. Despite a recent observation suggesting that low-carbohydrate diets were associated with higher EE,^47^ we did not find any significant associations for macronutrient composition of the diet in NDNS.

This study has several notable strengths. First, the NDNS is nationally representative and with no observed selection bias for the DLW subsample; therefore, the estimates for TEE and PAEE can serve as national reference values for this period. Second, DLW is the gold-standard method for measuring EE during free-living conditions. Third, our analyses include the main components and common expressions of EE, including both absolute and various relative measures, and within the limitations of the sample also reasonable stratification and multivariable adjustment analyses to test the robustness of observed differences across specific population subgroups.

This study also has some limitations. The study as a whole is not large, with only 770 individuals included in the present analyses. In addition, the majority of the sample came from England, with very few participants included in certain subgroup analyses. The generalizability of these small groups to the wider Northern Irish, Scottish and Welsh populations is therefore less certain. It is also possible that non-participating households may differ from participating households. Another limitation is that data are cross-sectional and effectively snapshot assessments taken at relatively short time intervals between 2008 and 2015, which, with this sample size, is unlikely to be sufficient to detect secular trends even if they truly occurred in the UK over this time period; given the slight increase in national obesity levels in the same period,^48^ we suspect that absolute TEE levels may have also increased but that relative EE levels may have decreased in line with the observed associations with such indicators in our study. Finally, some misclassification of the habitual diet cannot be ruled out, owing to only sampling 4 days of intake and using estimated portion sizes, rather than weighed quantities, but the method provides similar estimates of intake as the commonly used repeat 24-hour recall method, both of which compare reasonably well with biomarkers, particularly when normalized for energy intake as we have done here.^17^^,^^49^^,^^50^

In conclusion, age, sex and body composition are the main determinants of human EE. Results from this nationally representative sample using gold-standard methodology may serve as reference values for other population studies.

## Supplementary Data

Supplementary data are available at *IJE* online.

## Funding

The authors were supported by the UK Medical Research Council (unit programme numbers. MC_UU_12015/1, MC_UU_12015/3, U105960371) and the NIHR Biomedical Research Centre in Cambridge (IS-BRC-1215–20014). T.L. was funded by the Cambridge Trust.

## Supplementary Material

dyaa005_Supplementary_Data
