# Supplementary material for: Descriptive epidemiology of energy expenditure in the UK: findings from the National Diet and Nutrition Survey 2008–15
Source: Int J Epidemiol. 2020 Mar 19;49(3):1007–21. doi: 10.1093/ije/dyaa005 (PMC7394951; doi:10.1093/ije/dyaa005)
Supplement: dyaa005_Supplementary_Data [file dyaa005_supplementary_data.zip › dyaa005-Suppl_Data/ije-2019-02-0218-File029.pdf]

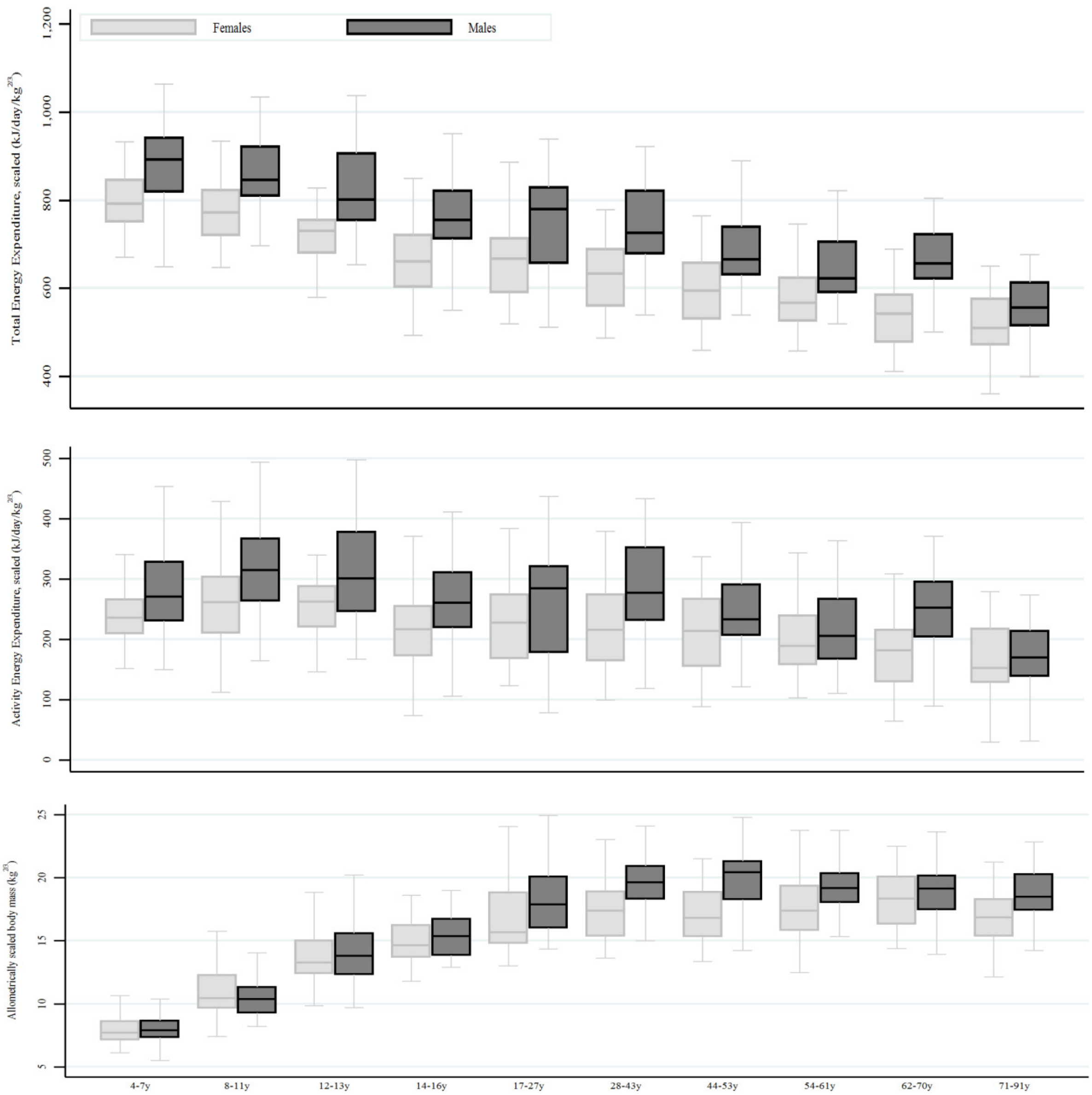

Figure S2. Allometrically scaled Total and Physical Activity-related Energy Expenditure per kg$^{2/3}$ total body mass by age (approximate deciles) and sex groups (Females= light grey; Males= dark grey). Bottom panel shows stratified allometrically scaled body mass.
