# Supplementary material for: Descriptive epidemiology of energy expenditure in the UK: findings from the National Diet and Nutrition Survey 2008–15
Source: Int J Epidemiol. 2020 Mar 19;49(3):1007–21. doi: 10.1093/ije/dyaa005 (PMC7394951; doi:10.1093/ije/dyaa005)
Supplement: dyaa005_Supplementary_Data [file dyaa005_supplementary_data.zip › dyaa005-Suppl_Data/ije-2019-02-0218-File027.pdf]

Supplement Table S1: Sensitivity analysis modelling Physical Activity Energy Expenditure per kg fat-free mass from stratifying variables (mutually adjusted)

Outcome: PAEE (kJ / day / kg FFM)

	Females		Males	
	PAEE (kJ / day / kg FFM)	C.I.	PAEE (kJ / day / kg FFM)	C.I.
**Model 1**				
**Age**				
4-10y	Reference		Reference	
11-15y	-15.3***	-23.8; -6.9	-10.6**	-20.2; -0.9
16-49y	-23.2***	-31.0; -15.4	-36.2***	-45.3; -27.2
50-64y	-25.0***	-33.3; -16.6	-43.3***	-52.7; -33.9
65-91y	-37.6***	-46.2; -29.0	-55.0***	-65.2; -44.8
**Year of Study**				
2008-2011	Reference		Reference	
2012-2015	0.8	-4.4; 5.9	-1.7	-7.8; 4.5
**Season**				
Spring	1.1	-2.5; 4.7	4.7**	0.6; 8.8
Winter	1.5	-2.2; 5.1	-0.2	-4.6; 4.3
**Region**				
South England	Reference		Reference	
North England	-1.7	-7.6; 4.2	-0.9	-8.0; 6.2
Scotland, Wales, Northern Ireland	5.6*	-1.0; 12.2	3.8	-4.2; 11.9
**FMI Category**				
1st Tertile	Reference		Reference	
2nd Tertile	-5.5*	-11.8; 0.9	-4.0	-11.3; 3.3
3rd Tertile	-11.9***	-18.5; -5.3	-8.3**	-15.7; -0.9
Constant	114.3***	107.0; 121.7	128.0***	118.7; 137.2
**Model 2 (with BF% instead of FMI)**				
F: <30% M: <25%	Reference		Reference	
F: 30-40% M: 25-35%	-7.1*	-14.5; 0.2	-6.1	-13.5; 1.3
F: >40% M: >35%	-17.9***	-25.8; -10.0	-9.7**	-18.3; -1.1

PAEE: Physical Activity Energy Expenditure; FFM, fat-free mass; FMI, fat mass index; BMI, body mass index; BF%, bodyfat %.
95% confidence intervals in parentheses
*** p<0.01, ** p<0.05, * p<0.1
